# Evaluation of a Brain Acetylcholinesterase Extraction Method and Kinetic Constants after Methyl-Paraoxon Inhibition in Three Brazilian Fish Species

**DOI:** 10.1371/journal.pone.0163317

**Published:** 2016-09-21

**Authors:** A. P. Freitas, C. R. Santos, P. N. Sarcinelli, M. V. Silva Filho, R. A. Hauser-Davis, R. M. Lopes

**Affiliations:** 1 Centro de Estudos da Saúde do Trabalhador e Ecologia Humana (CESTEH), ENSP, FIOCRUZ, Rua Leopoldo Bulhões, 1480, 21041-210, Rio de Janeiro, RJ, Brasil; 2 Universidade Federal do Rio de Janeiro, Centro de Ciências da Saúde, Instituto de Bioquímica Médica Leopoldo de Meis, Av. Carlos Chagas Filho, 373, Bl. D, S. 05, Cidade Universitária, Cep: 21941-902, Rio de Janeiro, RJ, Brasil; 3 Laboratório de Comunicação Celular, Instituto Oswaldo Cruz, FIOCRUZ, Av. Brasil, 4365 – Manguinhos, Rio de Janeiro, RJ, Brasil; Weizmann Institute of Science, ISRAEL

## Abstract

Acetylcholinesterase (AChE) is an important enzyme in the control of the neuronal action potential and sensitive to organophosphate inhibition. Brain fish AChE is less sensitive to organophosphate inhibition than AChE from terrestrial animals, although this sensitivity is variable among species and has not yet been fully evaluated in fish species. In this setting, inhibition kinetic constants for progressive irreversible inhibition of brain acetylcholinesterase due to methyl-paraoxon exposure were determined in three fish species (*Mugil liza*, *Genidens genidens* and *Lagocephalus laevigatus*) and hen (*Gallus domesticus*). Enzyme extraction using a detergent was shown to be adequate, and samples presented activity inhibition in high substrate concentrations and suppression of inhibition by methyl-paraoxon in the presence of the substrate, similar to kinetic patterns from purified enzyme preparations. Catfish (*G*. *genidens*) AChE presented the highest sensitivity among the evaluated fish species (IC50 = 1031.20 nM ± 63.17) in comparison to *M*. *liza* and *L*. *laevigatus* (IC50: 2878.83 ± 421.94 and 2842.5 ± 144.63 nM respectively). The lower dissociation constant (*K*_d_ = 20.3 ± 2.95 μM) of catfish AChE showed greater enzyme affinity for methyl-paraoxon, explaining this species higher sensitivity to organophosphates. Hen AChE presented higher *k*_*i*_ (900.57 ± 65.3 mM^-1^min^-1^) and, consequently, greater sensitivity to methyl-paraoxon, explained by a lower *K*_*d*_ (0.6 ± 0.13 μM). Furthermore, hen AChE did not differentiate between the propionylthiocholine and acetylthiocholine substrates, indicating easier access of methyl-paraoxon to the hen enzyme activity site. The results obtained herein indicate a suitable extraction of AChE and, despite different inhibition kinetic constants, demonstrate that fish AChE is less sensitive to methyl-paraoxon, probably due to reduced access to the catalytic center which provides greater enzyme substrate selectivity.

## Introduction

Acetylcholinesterase (EC 3.1.1.7, AChE) is a serine hydrolase enzyme found predominantly in muscle tissue and the nervous system, and controls the propagation of neuronal action potential through the hydrolysis of the neurotransmitter acetylcholine. This enzyme has a high affinity for choline esters, but is also able to hydrolyze other esters, such as organophosphate compounds (OP), at lower hydrolysis rate [1].

OPs are a diverse class of esters with a phosphorus atom in a phosphoryl (P = O) or thiophosphoryl (P = S) bond, initially designed as chemical warfare agents. Over the years, the use of these chemicals was expanded to many different purposes, such as pesticides in agriculture, ectoparasiticides in fish farming and drugs for medical disorders, resulting in a large anthropogenic dispersion in the environment, with potential to cause significant impacts on fish and other aquatic organisms [2–4]. The most recognized toxic effect of OPs is inhibition of AChE activity, occurring when the “oxon” (P = O) forms of OP, naturally present in the compound structure or acquired through biotransformation, phosphorylate a serine hydroxyl group present in the AChE catalytic center, leading to acetylcholine accumulation [5].

Regarding aquatic contamination by OPs, consequent fish impairment and selection of the most resistant species, resulting in fish fauna imbalance, is common. Indeed, the toxic effects of OPs may vary according to the impacted species, and previous results have demonstrated a number of specific differences among fish AChE sensitivity to methyl-paraoxon (dimethyl-4-nitrophenyl-phosphate, MP), an OP ([6–8]. Values of IC50-30min in different species ranged from 123 nM (*Prochilodus lineatus*) to 3108 nM (*Piaractus mesopotamicus*) both freshwater fishes, and from 455 nM (*Genidens genidens*) to 3340 nM (*Percophis brasiliensis*) for marine fishes. These differences in fish, 25-fold for freshwater and 7-fold for marine species, are unique among organisms belonging to the same taxonomic group. However, compared to other classes of vertebrates, fish show lower sensitivity to AChE inhibition by OP such as MP, while brain AChE from terrestrial vertebrates is much more sensitive to MP, with IC50-30min values ranging from 29 nM for *Rattus* (rat) to 37 nM for *Gallus* (chicken) [9].

These different AChE affinities regarding OPs are related to the kinetics between the enzyme and the inhibitor. In fact, AChE inhibition by methyl-paraoxon is described as being of a “progressively irreversible” kinetic character, since a reversible step occurs during the reaction between the enzyme and the inhibitor, forming a non-covalent intermediate, followed by an irreversible step that transforms this intermediate into an inactive complex. Equations for calculating the inhibition kinetic constants of this mechanism are available in the literature [10–12]. The velocity of the overall equation is governed by a constant *k*_*i*_, described by Main [12] as a “bimolecular reaction constant” incorporating an equation term related to the reversible reaction, the equilibrium constant (*k*_*d*_), and a term for the effective phosphorylation of the enzyme, the rate constant (*k*_*p*_).

Furthermore, many AChE molecular forms are anchored to the membrane, ensuring immobilization where enzyme activity is necessary, such as in the synapses and the motor end-plate. A difficulty concerning membrane proteins is that they are usually present in low levels, as well as the fact that the majority of these proteins are not generally soluble in aqueous solutions. To overcome this, solubilizing agents such as detergents may be applied [13]. However, most studies evaluate supernatant homogenates to test AChE activity without the use of detergents for AChE solubilization (Lopes *et al*. 2014). Thus, careful AChE preparation prior to enzyme activity evaluation should be observed in order to avoid nonspecific esterase activity and, therefore, incorrect interpretations of the results.

In Brazil, according to the Brazilian National Health Surveillance Agency (ANVISA), the pesticide market has expanded significantly over the last decade (190%), at a growth rate of more than double that of the global market (93%), placing Brazil at the top of the world ranking, since 2008 [14]. For instance, in 2013 approximately 905,000 tons of pesticides were sold in the country, with a total financial turnover of US$ 11.45 billion, as reported by the National Union of the Industry of Products for Plant Protection (SINDIVEG—http://sindiveg.org.br/). In addition, due to the recent Zika virus outbreaks in Brazil, a significant increase in the use of several pesticides, both against the mosquito larvae in the water or the adult vector, has been observed [15]. However, few studies evaluating the effects of MP in neotropical fishes, especially in Brazil, are available. This is of importance, since neotropical fauna is not well-characterized regarding biochemical responses to contamination [8, 16].

In this context, the present investigation aims to evaluate a brain AChE extraction method using a solubilizing agent, for the determination of the inhibition kinetics constants (IKC) after MP inhibition, and subsequent comparison of the calculated IKC of three neotropical Brazilian fish species to a higher vertebrate, hen (*Gallus domesticus)*, known as a highly sensitive species to this xenobiotic.

## Material and Methods

### Chemicals

Methyl-paraoxon and Triton X-100 was purchased from Riedel of Haën AG (Hanover). Acetyl, propionyl, butyrylthiocholine iodides and 5,5'-dithiobis-2-nitrobenzoic acid (DTNB) were obtained from Sigma Chemical Co. (St. Louis, MO). All reagents were of analytical grade.

### Animals and tissues

This work was approved by the Oswaldo Cruz Foundation (Fiocruz) Ethics Committee on the use of animals (approval number L0033/08). Fish samples were obtained from angling, following the legislation implemented by the Brazilian Institute of Environment and Renewable Natural Resources (IBAMA). *Genidens genidens* and *Lagocephalus laevigatus* specimens were obtained from artisanal fishermen at Itaipuaçu beach, Maricá, Rio de Janeiro, Brazil. Mullet (*Mugil liza*) were caught at two coastal lagoons in the northern area of Rio de Janeiro, as part of a monitoring project conducted by the Biochemistry Laboratory at the State University of Rio de Janeiro (UERJ), and kindly provided by Dr. Jayme da Cunha Bastos.

Fish were anaesthetized by exposure to an eugenol/ethanol solution 50% (v/v) for six minutes or until the position of fish in the water became random, with prevalence of the dorsal position and no response to external stimuli [17]. The animals were immediately euthanized by spine severing and processed in conformity with the ethical principles of animal experimentation, elaborated by the Brazilian College for Animal Experimentation (COBEA) and in accordance with requirements of the National Institutes of Health guide for the care and use of Laboratory animals.

Hen (*Gallus domesticus)* samples (decapitated heads) were obtained from a slaughterhouse (“*Venda da Cruz*”) located in the city of São Gonçalo—RJ, and immediately frozen at -20°C. In the laboratory, the heads were thawed; the brains were dissected, washed with saline, blotted with filter paper and weighed.

### Brain AChE enzyme preparation

Whole brains were suspended in buffer (1 g of tissue plus 10 mL sodium phosphate 0.1 M, pH 7.5) and homogenized in an ice bath using an Ultra-Turrax homogenizer. The homogenate was then centrifuged (5,000*g*/30 min/5°C), and the soluble fraction was discarded. The pellet was then resuspended using the same volume of a 0.1 g% Triton X-100 in a 0.1 M sodium phosphate, pH 7.5, solution, and re-homogenized. After a second centrifugation (15,000*g*/90 min/5°C), the supernatant resulting from the pellet ressolubilization, or “solubilized AChE—AchEsol” [7], was collected, frozen, and stored at -20°C. All assays were conducted at most 72 hours after production of the supernatant fractions (AChEsol).

### Enzymatic assay

Brain AChE activity was assayed spectrophotometrically in 0.1 M sodium phosphate Triton X-100 0.1 g% pH 7.5 by a modification of Ellman’s method [18], as described by Silva Filho and colleagues [7]. The specificity of AChE for substrates (P%) was calculated by the ratio of AChE activity when using propionylthiocholine as substrate divided by AChE activity when using acetylthiocholine: *P*%, [(activity with ProScol/activity with AceScol) x 100] (%) [6].

### Methyl-paraoxon solutions

MP stock solutions were prepared in methanol and their stability was verified by measuring the 4-nitrophenol (PNP) produced by alkaline hydrolysis degradation using a molar extinction coefficient of 18,600M^-1^.cm^-1^ at 401 nm [5]. Residual PNP in the pesticide solution was determined by the Y-intercept of the linear regression from a plot of absorbance versus time for the firsts 20s of alkaline hydrolysis. Degradation rates in the form of residual PNP were tolerated up to 5% [7, 8].

### Kinetic analyses

Inhibition kinetic constants (IKC) were obtained using the methods described by Kemp and Wallace [9] and Carr and Chambers [19], and by the equations proposed by Kitz and Wilson [10] and Main [12]. The details and rationale involving the kinetic analysis procedures described herein have been reported previously [7] and applied to other studies [6, 8]. Briefly, for IKC calculations two main conditions are required: (1) no enzyme inhibition in the presence of MP and 1.875mM acetylthiocholine; (2) the lowest concentrations of MP must be significantly higher than those of the enzyme. The first condition is established by the absence of inhibition of the enzyme from the addition of the MP after the addition of substrate. The second is established when a linear regression occurs between ln([*E*_*r*_]/[*E*_*0*_]) and incubation time, indicating a first-order reaction between the enzyme and inhibitor.

Enzyme inhibition was evaluated by the incubation of 50 μL MP solutions in methanol, containing different MP concentrations (ranging from 7 to 208 μM for *Mugil liza*, from 7 to 34 μM for *Genidens genidens*, from 7 to 41 μM for *Lagocephalus laevigatus* and from 0.069 to 0.339 μM for *Gallus domesticus*), with 0.45 mL of the enzyme solution containing around 5–6 mU of AChE in sodium phosphate buffer, pH 7.5, plus 0.1 g% Triton X-100. After 1, 2, 3, 4 and 5 min of incubation, enzyme inhibition was stopped by adding 0.5 mL of 0.64 mM DTNB-3.75 mM acetylthiocholine (a 2X normal concentration) in sodium phosphate buffer 0.1 M, pH 7.5, plus 0.1 g% of Triton X-100 prepared prior to use. The residual enzyme activities [*E*_*r*_] were evaluated. Control enzyme activities [*E*_*0*_] were measured by adding the pesticide after the substrate. Regressions were calculated with MP concentrations that resulted in 10%-90% enzyme inhibitions, as indicated by Kemp and Wallace [9]. With low inhibitor concentrations (e.g. 5μM of MP), resulting in less than 10% of inhibition of AChE obtained from mullet, it was very difficult to obtain a good regression, with R^2^ higher than 0.95. With high inhibitor concentrations (e.g. 208μM of MP), when inhibition was higher than 90%, the *Er* results were completely out of the straight line for *k*_app_ determination (R^2^ < 0.95).

One Unit of AChE (U) is the amount of enzyme that forms one μmol of products per minute. The Coefficient of Determination (R^2^) was used to express the linearity of the regressions, assuring that calculated constants did not depend on the amount of pesticide [20]. The linear regressions of ln([*E*_*r*_]/[*E*_*0*_]) over time (plot) and linear regressions of double-reciprocals plots (replots) were considered only when R^2^ was higher than 0.95. As a measure of enzyme sensitivity to MP, the IC50 was calculated for each species for 30 min of incubation using the following equation $\text{IC}50-30\text{min}=\frac{-\text{ln}(0.5)}{\left(k_{i}*30\right)}$ [21].

## Results and Discussion

### Substrate concentration effects on mullet and hen AChE activity

**Fig 1:**
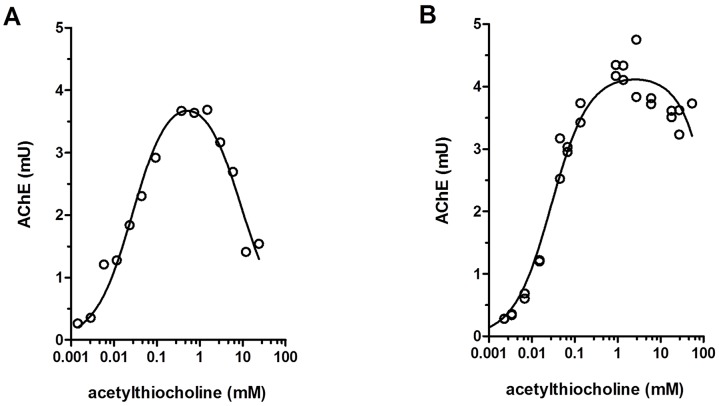
Substrate inhibition of brain AChE from mullet (*Mugil liza*) (A) and hen (*Gallus*) (B). Each point represents an AChE assay for enzymes pooled from three different animals. Curves were fitted as proposed by Radic *et al*. [22] for the inhibition of AChE by excessive substrate: R^2^ = 0.9618 for mullet enzyme; R^2^ = 0.9699 for the chicken enzyme.

**Fig 2:**
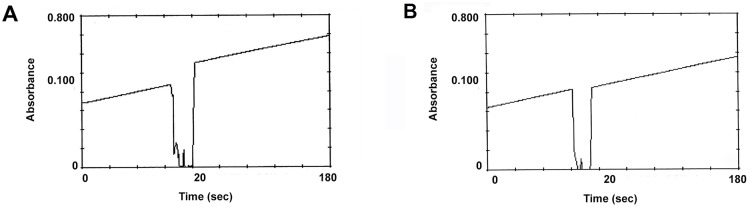
Competitive pattern of brain AChE inhibition by methyl-paraoxon (MP). The graphs show the progress of the AChE assays by Ellman’s method for mullet (*Mugil liza*) (A) and hen (*Gallus domesticus*) (B) enzymes. After 1 minute of enzyme reaction, a final concentration of 50μM (A) and 0.5μM (B) of MP was obtained by adding the same volume of the MP methanol solution, following 3 minutes of enzyme reaction. Initial enzyme activities were prepared the same way for the two enzymes and inhibitor concentrations were prepared to allow for 80% inhibition after 2 minutes of incubation without any substrate for the two enzymes. The valleys represent the moments of MP addition. The higher absorbance after the addition of MP for *M*. *liza* enzyme assay (A) was due to the presence of 4-nitrophenol in the MP solution (maximum of 5%).

Inhibitions by excess substrate (Fig 1) and the complete blocking of MP inhibition in the presence of substrate (Fig 2) were used to guarantee adequate AChE preparation and pre-conditions of the progressive irreversible inhibition mechanism for subsequent IKC determinations. *Gallus domesticus* brain AChE, an example of a highly sensitive enzyme, was compared to brain AChE from fish (*Mugil liza*), less sensitive. Curves were fitted as proposed by Radic *et al*. [22] for inhibition of AChE by excessive substrate. AChE from both species displayed similar activity patterns, with higher enzyme activity with increasing substrate concentrations, and enzyme saturation around 1 mM of substrate (Fig 1).

Enzyme activity towards acetylthiocholine with progressive inhibition in excess of substrate is described as characteristic AChE property [1, 23] indicating adequate enzyme extraction. Furthermore, the substrate prevented AChE inhibition by MP (Fig 2), demonstrated by the linearity throughout the 3 minutes of the enzyme assay for the determination of AChE activity.

These results demonstrated the predicted activity inhibition in high substrate concentration [22], and the complete suppression of inhibition by MP in the presence of the substrate acetylthiocholine suggesting a competitive inhibition mechanism. The extracted AChE preparations showed properties similar to the purified enzyme preparations and were considered suitable for inhibition assays.

### AChE activity inhibition at three MP concentrations

**Fig 3:**
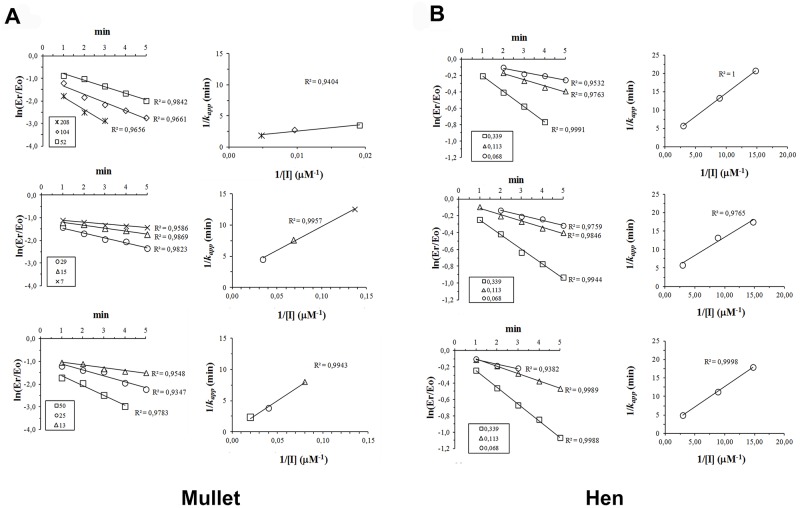
*k*_*app*_ determination plots (graphs on the left) and replots (graphs on the right) for the inhibition kinetic constants (IKC) for methylparaoxon (MP) inhibition of brain AChE from three *M*.*liza* (A) and three *G*. *domesticus* (B). The boxes show methylparaoxon (MP) concentrations (μM). Each point for the *k*_*app*_ determination represents an individual enzyme assay.

Different MP concentrations were evaluated for the IKC calculations in the AChE inhibition assay and for the animal comparisons (Fig 3).

For all replots, a minimum of 10 and a maximum of 90% AChE inhibition was observed. Higher inhibitions led to results out of the range of the plotted straight line. For all inhibitor concentrations tested herein, with the exception of one hen assay, *k*_*app*_ linearity (R^2^ > 0.95) was observed, indicating adequate MP concentrations for IKC calculations, according to Kemp and Wallace [9].

### k_app_ replots and IKC calculations for the three fish species and hen

**Fig 4:**
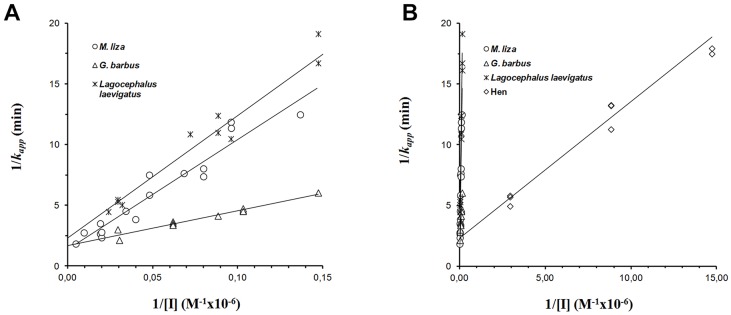
Replots of the *k*_*app*_ for the IKC determinations of brain AChE from mullet (*Mugil liza*), catfish (*Genidens genidens*), puffer fish (*Lagocephalus laevigatus*) (4A), and hen (*Gallus*) (4B) inhibited with methyl-paraoxon (MP). Each point represents an individual *k*_*app*_ determination.

Similar *k*_*app*_ were observed for all investigated species, both fish and hen. Linear regressions presented different slopes, with the exception of *L*. *laevigatus* and *M*. *liza*, that showed similar slopes (Fig 4).

The more vertical the lines, the lower the sensitivity of brain AChE to inhibition by methyl-paraoxon, since the slopes represent the inverse of the *k*_*i*_. Different regression convergence points may indicate different IKC values, and parallel lines denote identical sensitivity to the inhibitor (same *k*_*i*_), which is only possible with proportional increments or decrements in *k*_*p*_ and *K*_*d*_ values (Silva *et al*., 2004). As displayed in Fig 4A, catfish (*G*. *genidens)* AChE presented higher sensitivity to MP inhibition when compared to the other evaluated fish species. This was confirmed by the IKC values and the calculated IC50 for this species (Table 1).

**Table 1:** Brain acetylcholinesterase kinetics constants for mullet, catfish, puffer fish, and hen.

Species	*k*_*i*_	*k*_*p*_	*k*_*d*_	IC50	P%
*Mugil liza*	10.77 ± 0.69	1.64 ± 0.6	159.4 ± 59.55	2878.83 ± 421.94	17
*Genidens genidens*	35.28 ± 1.6	0.71 ± 0.1	20.3 ± 2.95	1031.20 ± 63.17	37
*Lagocephalus leavigatus*	9.31 ± 0.76	0.55 ± 0.08	62.6 ± 14.74	2842.5 ± 144.63	30
*Gallus domesticus*	900.57 ± 65.3	0.47 ± 0.1	0.6 ± 0.13	25.67 ± 1.76	103

*k*_*i*_: Bimolecular reaction constant (mM^−1^ min^−1^); *k*_*p*_: phosphorylation rate constant (min^−1^); *K*_*d*_: dissociation constant of enzyme-inhibitor complex (EI) (μM) or affinity constant for methyl-paraoxon; IC50: concentration of methyl-paraoxon (nM) that produced 50% inhibition of brain AChE activity in 30 min, estimated using the *k*_*i*_; P(%): AChE activity with substrate propionylthiocholine expressed as % of the activity with substrate acetylthiocholine.

The greater sensitivity of Catfish (*G*. *genidens*) AChE to inhibition by MP is revealed by the *k*_*i*_ value, three times higher than for the other evaluated fishes. However, puffer fish (*L*. *laevigatus)*, and catfish (*G*. *genidens)* showed similar *k*_*p*_ values. These differences must, therefore, be related to differences in AChE affinity to the inhibitor MP. Indeed, the catfish enzyme dissociation constant (*K*_*d*_) was about three times lower than puffer fish (*L*. *laevigatus)*, and 8 times lower than mullet (*M*. *liza)*, confirming the fact that catfish AChE sensitivity to MP is higher as a consequence of a higher enzyme affinity for the inhibitor, expressed as *K*_*d*_ differences [9, 19, 21, 24].

Similar AChE enzyme *k*_*p*_ values were observed for catfish (*G*. *genidens*), puffer fish (*L*. *laevigatus*), and hen (*Gallus*), with a slightly higher value for mullet (*M*. *liza)*. However, *k*_*i*_ and *K*_*d*_ values differed significantly between animals. Hen AChE presented higher sensitivity to MP, as indicated by a lower IC50 value.

Brain AChE from mullet (*M*. *liza)* and puffer fish (*L*. *laevigatus)* showed about the same sensitivity (Table 1 *k*_*i*_) to MP, as indicated by the parallel lines displayed in Fig 4. The *k*_*p*_ and *K*_*d*_ values for brain mullet and puffer fish AChE (Table 1) were different but both constants vary in the same order of magnitude between these fish species. Since *k*_*i*_ is the slope of these contants (*k*_*p*_/*K*_*d*_) this leads to similar *k*_*i*_ and, hence, similar AChE sensitivity. Hen (*Gallus domesticus*) AChE IKC showed a much higher *k*_*i*_ value (Table 1), and thus, greater sensitivity to MP, as expressed by the much lower IC50 value. This greater susceptibility to OP is explained by the lower *K*_*d*_ value and, therefore, hen AChE presented a much higher affinity to MP when compared to fish brain AChE.

AChE sensitivity to MP, expressed as IC50, and substrate specificity, expressed as P% (Table 1), confirm the hypothesis that AChE from hen offers easier MP access to its catalytic center. The more sensitive hen brain AChE (with the lowest IC50 value), showed the highest P%, indicating steric differences between brain AChE structures of hen and fishes. A more sensitive enzyme to MP, such as brain AChE from hen, may show less substrate specificity. Hen brain AChE activity does not differentiate between the two evaluated substrates, with P% around 100%. Choline substrates have been shown to present a positive charge, which is lacking in MP [7, 25]. The results obtained herein, thus, suggest that a larger catalytic center may be required for increased AChE substrate sensitivity, as proposed previously by Kemp and Wallace [9].

The low variability of the catalytic center residues indicate that the enzyme mechanism was subjected to a strong selection under natural conditions, suggesting that sensitivity variability is due to affinity differences based on changes in peripheral residues of aromatic gorge or anionic center, and not based on catalytic center residues. This is further supported by the higher variability of the *K*_*d*_ observed for brain AChE in some fish and vertebrates such as hen and rat, with similar *k*_*p*_ values [7]. In light of the results presented herein, terrestrial vertebrate brain AChE enzymes appear to be much more sensitive to inhibitors and much less specific to artificial substrates than fish brain AChE.

## Conclusions

The results of the present study demonstrate that the insoluble fraction of brain AChE with detergents provides suitable enzyme preparations with a simple method for accurate enzyme assays eliminating complex purification steps. Furthermore, this study demonstrated that terrestrial vertebrate brain AChE enzymes appear to significantly more sensitive to inhibitors and much less specific to artificial substrates than fish brain AChE that demonstrated low affinity, as expressed by the high *K*_*d*_ values. This may be due to a reduced access to the catalytic center, providing greater substrate selectivity for fish brain AChE.
